# Improved anticancer drug response prediction in cell lines using matrix factorization with similarity regularization

**DOI:** 10.1186/s12885-017-3500-5

**Published:** 2017-08-02

**Authors:** Lin Wang, Xiaozhong Li, Louxin Zhang, Qiang Gao

**Affiliations:** 1 School of Computer Science and Information Engineering, Tianjin University of Science and Technology, Tianjin, 300457 China; 2 Department of Mathematics, National University of Singapore, Singapore, 119076 Singapore; 3 Key Lab of Industrial Fermentation Microbiology, Ministry of Education & Tianjin City, College of Biotechnology, Tianjin University of Science and Technology, Tianjin, 300457 China

**Keywords:** Anticancer drug response prediction, Matrix factorization, Precision cancer medicines, Drug repositioning

## Abstract

**Background:**

Human cancer cell lines are used in research to study the biology of cancer and to test cancer treatments. Recently there are already some large panels of several hundred human cancer cell lines which are characterized with genomic and pharmacological data. The ability to predict drug responses using these pharmacogenomics data can facilitate the development of precision cancer medicines. Although several methods have been developed to address the drug response prediction, there are many challenges in obtaining accurate prediction.

**Methods:**

Based on the fact that similar cell lines and similar drugs exhibit similar drug responses, we adopted a similarity-regularized matrix factorization (SRMF) method to predict anticancer drug responses of cell lines using chemical structures of drugs and baseline gene expression levels in cell lines. Specifically, chemical structural similarity of drugs and gene expression profile similarity of cell lines were considered as regularization terms, which were incorporated to the drug response matrix factorization model.

**Results:**

We first demonstrated the effectiveness of SRMF using a set of simulation data and compared it with two typical similarity-based methods. Furthermore, we applied it to the Genomics of Drug Sensitivity in Cancer (GDSC) and Cancer Cell Line Encyclopedia (CCLE) datasets, and performance of SRMF exceeds three state-of-the-art methods. We also applied SRMF to estimate the missing drug response values in the GDSC dataset. Even though SRMF does not specifically model mutation information, it could correctly predict drug-cancer gene associations that are consistent with existing data, and identify novel drug-cancer gene associations that are not found in existing data as well. SRMF can also aid in drug repositioning. The newly predicted drug responses of GDSC dataset suggest that mTOR inhibitor rapamycin was sensitive to non-small cell lung cancer (NSCLC), and expression of AK1RC3 and HINT1 may be adjunct markers of cell line sensitivity to rapamycin.

**Conclusions:**

Our analysis showed that the proposed data integration method is able to improve the accuracy of prediction of anticancer drug responses in cell lines, and can identify consistent and novel drug-cancer gene associations compared to existing data as well as aid in drug repositioning.

**Electronic supplementary material:**

The online version of this article (doi:10.1186/s12885-017-3500-5) contains supplementary material, which is available to authorized users.

## Background

Patients suffering from the same cancer may differ in their responses to a specific medical treatment. Precision cancer medicines aim to decipher the cause of a given patient’s cancer at the molecular level and then tailor treatment to address that patient’s cancer progression [1]. Identification of predictive biomarker for drug sensitivity in individuals is the key that will promote precision cancer medicine [2]. Human cancer cell lines, compared to human or animal model, have been popular to study the cancer biology and drug discovery through facile experimental manipulation. Several large-scale high-throughput screenings have catalogued genomic and pharmacological data for hundreds of human cancer cell lines, respectively [3–6]. Development of computational methods that link genomic profiles of cancer cell lines to drug responses can facilitate the development of precision cancer medicines, for which the identified genomic biomarkers can be used to predict anticancer drug response [7, 8].

Machine learning algorithms such as elastic net regularization and random forests were used to search for genomic biomarkers of drug sensitivity in cancer cell lines for individual drugs [3–5, 9, 10]. Recently, Seashore-Ludlow et al. developed a cluster analysis method integrating information from multiple drugs and multiple cancer cell lines to identify genomic biomarkers [6]. Geeleher et al. improved genomic biomarker discovery by accounting for variability in general levels of drug sensitivity in pre-clinical models [11]. In contrast to genomic biomarker identification, some research works focused on drug response prediction. Before-treatment baseline gene expression levels and in vitro drug sensitivity in cell lines were used to predict anticancer drug responses [12, 13]. Daemen et al. used least square-support vector machines and random forests algorithms integrating molecular features at various levels of the genome to predict drug responses from breast cancer cell line panel [14]. Menden et al. predicted drug responses using neural network where each drug-cell line pair integrated genomic features of cell lines with chemical properties of drugs as predictors [15]. Ammad-ud-din et al. applied kernelized Bayesian matrix factorization (KBMF) method to predict drug responses in GDSC dataset [16]. The method utilized genomic and chemical properties in addition to drug target information. Liu et al. used drug similarity network and cell similarity network to predict drug response, respectively, meaning that predictions were done twice separately. Then the final prediction is obtained as a weighted average of the two predictions based on dual-layer network (DLN) [17]. Cortés-Ciriano et al. proposed the modelling of chemical and cell line information in a machine learning model such as random forests (RF) or support vector regression to predict the drug sensitivity of numerous compounds screened against 59 cancer cell lines from the NCI60 panel [18]. Although various methods have been developed to computationally predict drug responses of cell lines, there are many challenges in obtaining accurate prediction.

Based on the fact that similar cell lines and similar drugs exhibit similar drug responses [17], here we propose a similarity-regularized matrix factorization (SRMF) method for drug response prediction which incorporates similarities of drugs and of cell lines simultaneously. To demonstrate its effectiveness, we applied SRMF to a set of simulated data and compared it with two typical similarity-based methods: KBMF and DLN. The evaluation metrics include Pearson correlation coefficient (PCC) and root mean square error (RMSE). The results showed that SRMF performed significantly better than KBMF and DLN in terms of drug-averaged PCC and RMSE. Moreover, we applied SRMF to GDSC and CCLE drug response datasets using ten-fold cross validation which showed that the performance of SRMF significantly exceeded other existing methods, such as KBMF, DLN and RF. We have also applied SRMF to infer the missing drug response values in the GDSC dataset. Even though the SRMF model does not specifically model mutation information, it correctly predicted the associations between EGFR and ERBB2 mutations and sensitivity to lapatinib that targets the product of these genes. Similar fact was observed with predicted response of CDKN2A-mutated cell lines to PD-0332991. Furthermore, by combining newly predicted drug responses with existing drug responses, SRMF can identify novel drug-cancer gene associations that do not exist in the available data. For example, MET amplification and TSC1 mutation are significantly associated with c-Met inhibitor PHA-665752 and mTOR inhibitor rapamycin, respectively. Finally, the newly predicted drug responses can guide drug repositioning. The mTOR inhibitor rapamycin is sensitive to non-small cell lung cancer (NSCLC) based on newly predicted drug responses versus available observations. Besides, expression of AK1RC3 and HINT1 were identified as biomarkers of cell line sensitivity to rapamycin.

## Methods

### Data and preprocessing

We firstly used the data from the Genomics of Drug Sensitivity in Cancer project consisting of 139 drugs and a panel of 790 cancer cell lines (release-5.0). Experimentally determined drug response measurements were determined by log-transformed IC50 values (the concentration of a drug that is required for 50% inhibition in vitro, given as natural log of μM). Notably, a lower value of IC50 indicates a better sensitivity of a cell line to a given drug. In addition, cell lines were characterized by a set of genomic features. We selected the 652 cell lines for which both drug response data and gene expression were available. Furthermore, we focused on the 135 drugs for which SDF format (encoding the chemical structure of the drugs) were available from the NCBI PubChem Repository. Then PubChem fingerprint descriptors were computed using the PaDEL software [19]. The resulting drug response matrix of 135 drugs by 652 cell lines has 88,020 entries, out of which 17,344 (19.70%) are missing and 70,676 are known. For a pair of drugs, the similarity between their fingerprints was measured by the Jaccard coefficient. The cell line similarities, on the other hand, were calculated based on their gene expression profiles, and Pearson correlation coefficient was used to compute the profile similarity between two cell lines.

The data from the Cancer Cell Line Encyclopedia consists of 24 drugs and a panel of 1036 human cancer cell lines. Drug sensitivity data were summarized by activity area (the area over the drug response curve). Notably, the higher the activity area value, the better the sensitivity. We selected the 491 cancer cell lines for which both drug sensitivity measures and gene expression profile data were available. There are 23 drugs having PubMed SDF files from which we can obtain drug chemical structures. The resulting drug response matrix of 23 drugs by 491 cell lines has 11,293 entries, out of which 423 (3.75%) are missing and 10,870 are known.

### Problem formulation

**Fig. 1:**
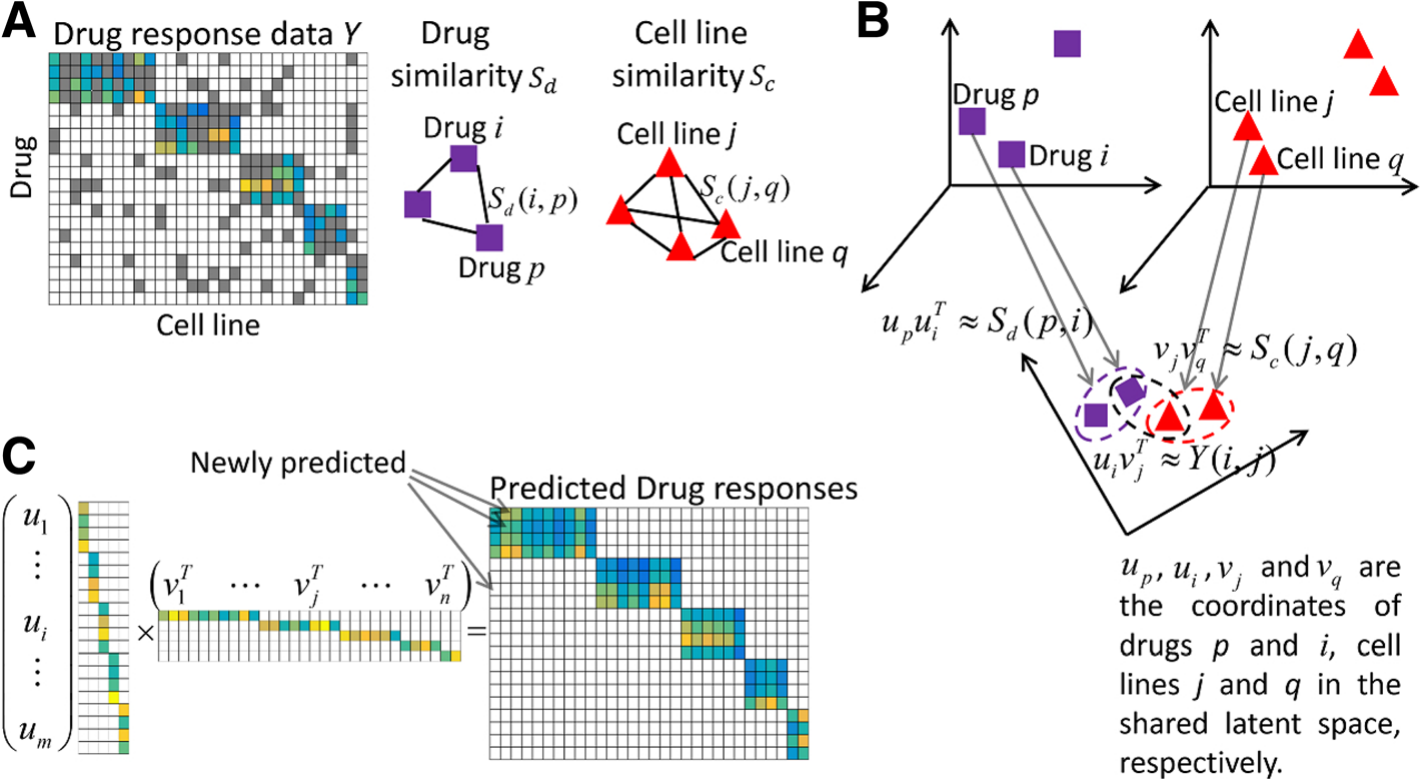
The framework of drug response prediction method SRMF. **a** The input data for SRMF includes the available drug responses (such as active area values) in cancer cell lines versus the unknown values marked as *grey*, chemical structure-based drug similarity and gene expression profile-based cell line similarity. **b** Rationale for the matrix factorization approach. Drugs and cell lines are mapped into a shared latent space with a low dimensionality. Furthermore, the associations among drugs and cell lines are described using the inner products of their coordinates in the shared latent space. **c** SRMF computes the coordinates of drugs and cell lines *U* and *V* in the shared latent space, which are used to reconstruct drug response matrix including the newly predicted drug responses

In this article, we applied a powerful matrix factorization framework to predict anticancer drug responses in cell lines (Fig. 1). Similar framework has been adopted to predict drug targets [20]. The primary idea is to map *m* drugs and *n* cell lines into a shared latent space, with a low dimensionality *K*, where *K* ≪ min (*m*, *n*). The properties of a drug *d*_*i*_ and a cell line *c*_*j*_ are described by two latent coordinates *u*_*i*_ and *v*_*j*_(*K* dimensional row vectors), respectively. As to drug response matrix *Y*, we aimed to approximate each known response value of drug*d*_*i*_ for cell line *c*_*j*_ via their latent coordinates which can be our objective function:

1 $$ \underset{U,V}{ \min}\Big|{\left|W\cdot \left(Y-{UV}^T\right),\Big|\right|}_F^2, $$

where *W* is a weight matrix, in which *W*_*ij*_ = 1 if *Y*_*ij*_ is a known response value; otherwise *W*_*ij*_ = 0, *W* ⋅ *Z* denotes the Hadamard product of two matrices *W* and *Z, U* and *V* are two matrices containing *u*_*i*_ and *v*_*j*_ as row vectors, respectively, and ∣|⋅|_*F*_ is the Frobenius norm*.*

Then to avoid overfitting of *U* and *V* to training data, L2 (Tikhonov) regularization was imposed to the latent variables *U* and *V*.

2 $$ \underset{U,V}{ \min}\Big|{\left|W\cdot \left(Y-{UV}^T\right)\Big|\right|}_F^2+{\lambda}_l\left(|{\left|U,\Big|\right|}_F^2+|{\left|V,\Big|\right|}_F^2\right), $$

Furthermore, prior knowledge on drugs and cell lines is very useful and valuable to decipher the global structure of drug-cell line response data. Based on the results that similar cell lines and similar drugs exhibit similar drug responses [17], we proposed to exploit the drug similarity and cell line similarity to further improve the drug response prediction accuracy. The primary idea of exploiting the drug (cell line) similarity information for drug response prediction is to minimize the differences between similarity of two drugs (cell lines) and that of them in the latent space. These objectives can be achieved by minimizing the following objective functions (3) and (4):

3 $$ \Big|{\left|{S}_d-{UU}^T,\Big|\right|}_F^2, $$

4 $$ \Big|{\left|{S}_c-{VV}^T\Big|\right|}_F^2, $$

where *S*_*d*_ and *S*_*c*_ are drug similarity matrix and cell line similarity matrix, respectively.

The final drug response prediction model can be formulated by considering the drug response matrix as well as the similarity of drugs and cell lines. By plugging Eqs (3) and (4) into Eq. (2), the proposed SRMF model is formulated as follows:

5 $$ \underset{U,V}{ \min}\left|{\left|W\cdot \left(Y-{UV}^T\right),\Big|\right|}_F^2+{\lambda}_l\left(|{\left|U,\Big|\right|}_F^2+|{\left|V,\Big|\right|}_F^2\right)+{\lambda}_d\right|{\left|{S}_d-{UU}^T,\Big|\right|}_F^2+{\lambda}_c\Big|{\left|{S}_c-{VV}^T,\Big|\right|}_F^2. $$

### The SRMF algorithm

Since the objective function (5) is not convex with respect to variables *U* and *V*, we searched for the local minimum instead of the global minimum by an alternating minimization algorithm. The algorithm which was deduced detailedly in Additional file 1 updates variables *U* and *V* alternately. We provided this algorithm in the following, and the software can be freely downloaded from the website (https://github.com/linwang1982/SRMF).
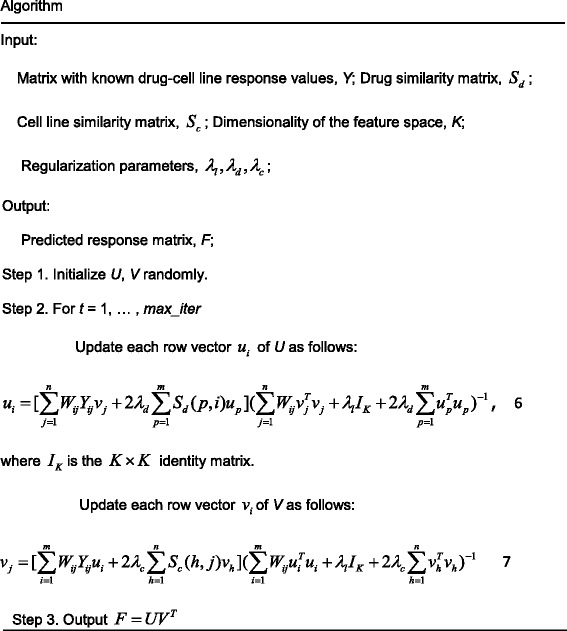


### Measurements of prediction performance

By accounting for variability in sensitive ranges of drugs, the correlation between observed and predicted response values for all drug response entries may overestimate the prediction performance [17]. Here, we focused on evaluation metrics for individual drugs, including Pearson correlation coefficient (PCC) and root mean squared error (RMSE) for each drug [17]. RMSE is computed as follows,

8 $$ RMSE(D)=\sqrt{\frac{\sum_C{\left(\mathrm{R}\left(D,C\right)-\widehat{R}\left(D,C\right)\right)}^2}{n}} $$

where *n* is the number of cell lines with known response values for drug *D*, R(*D*, *C*) and $\widehat{R}\left(D,C\right)$ are observed and predicted response values for drug *D* versus cell line C, respectively. Moreover, drug-averaged PCC and RMSE are computed as the average PCC and RMSE over all drugs.

There is compelling evidence that the sensitive and resistant cell lines of each individual drug are more valuable to decipher mechanisms of drug actions, we also care about PCC and RMSE from sensitive and resistant cell lines for each drug, and they were denoted as PCC_S/R and RMSE_S/R, respectively. Here, for each drug the logIC50 (activity area) were split into quartiles, with cell lines in the first and fourth representing drug-sensitive (−resistant) and –resistant (−sensitive) cell lines, respectively, which was also performed for drug sensitive analysis of breast cancer cell lines [21]. Consequently, we have drug-averaged PCC_S/R and RMSE_S/R which are the average PCC_S/R and RMSE_S/R over all drugs, respectively.

### Experimental settings

The settings of the hyper-parameters of each method were as follows. For the matrix factorization based methods, including SRMF and KBMF, the low dimensionality *K* was set as 45 for GDSC dataset [16]. Moreover, as to SRMF, the drug response matrix was scaled in the way that its elements lie within the range [−1, 1] by dividing through the maximum absolute value of the matrix, so that the data range is similar with that of drug (cell line) similarity matrix, and the regularization parameters *λ*_*l*_ , *λ*_*d*_ , *λ*_*c*_ of SRMF were selected from{2^‐3^,  … , 2^2^}, {2^‐5^,  … , 2^1^, 0}and{2^‐5^,  … , 2^1^, 0}, respectively. In DLN, the decay parameters *σ* and *τ*were chosen from range of [0, 1] at 0.001 increments and 0.01 increments, respectively. The weight parameter *λ* was selected from range of [0, 1] at 0.01 increments [17]. For a prediction method, the most suitable hyper-parameters on different datasets are usually different. Thus, we adopted grid search to choose the optimal hyper-parameters for each drug response prediction method on each dataset. RF treated drug response prediction as a regression problem where each possible drug-cell line pair integrated genomic features of the cell line with chemical fingerprint features of the drug as predictors. For RF, genomic features of cell lines used the gene transcript levels for the 1000 genes display the highest variance across the cell line panel, and all fingerprint features with constant values across all drugs were removed [18].

## Results

### Similar cell lines are sensitive to similar drugs

**Fig. 2:**
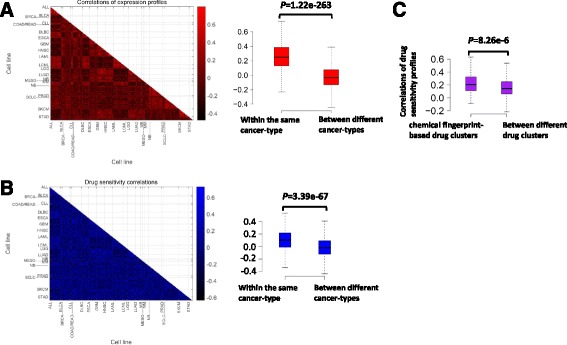
Similar cell lines respond similarly to the similar drugs. **a** Lower triangular matrix containing Pearson correlation between each pair of gene expression profiles of cell lines. The X-axis and Y-axis represent cell lines classified by their cancer-types (TCGA classification). *Box plots* show the correlations of gene expression within the same and between different cancer-types. **b** *Lower triangular* matrix containing Pearson correlation between each pair of drug sensitivity profiles of cell lines. *Box plots* show the correlations of drug sensitivity within the same and between different cancer-types. **c** *Box plots* show the correlations of sensitivity profiles across cell lines within the same and between different drug clusters. The drugs were hierarchically clustered according to the similarity of their chemical fingerprints. The one-sided Mann–Whitney *U* test was used to measure the statistical difference between two groups

We calculated the Pearson correlation between each pair of gene expression profiles of cell lines after normalizing gene expression values across cell lines. As shown in Fig. 2a, gene expression correlations were significantly higher for cell lines within the same cancer type. This is in agreement with the tissue specificity of gene expression [22]. Furthermore, we calculated the Pearson correlation coefficient of drug responses for each cell line pair after normalizing drug response values across cell lines. Figure 2b shows that drug sensitivity correlations were also significantly higher for cell lines within the same cancer-type. These results suggest that cell lines with similar gene expression profiles tend to be within the same cancer-type, which have similar responses for the same drug.

A hierarchical clustering of 135 drugs based on their chemical fingerprint features was performed (Additional file 2). Furthermore, we calculated the Pearson correlation between each pair of sensitivity profiles of drugs. Drug pairs within the same cluster of chemical fingerprints have significantly higher drug sensitivity correlations (Fig. 2c). This result depicts that drugs with similar chemical fingerprints show similar inhibitory effects on the same cell line.

### Simulation study

**Fig. 3:**
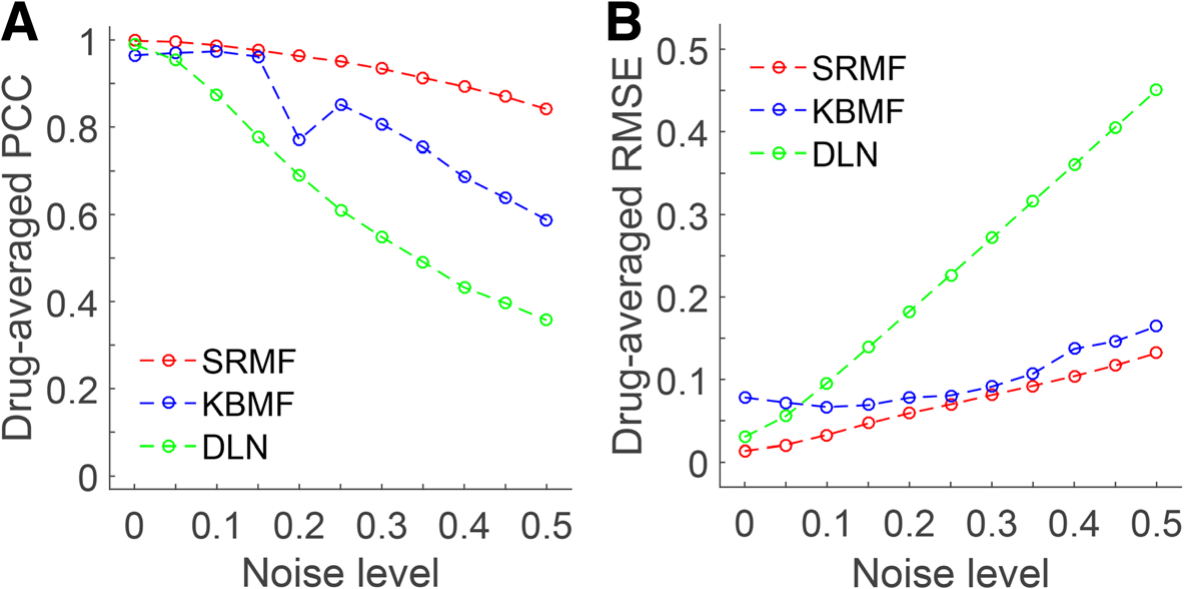
Evaluation of different prediction methods through simulations. We compared the performance of SRMF, KBMF and DLN for the estimation of target drug response. The dimensions of the simulation results are *m* = 100, *n* = 150. Details of the simulation methods are in Additional file 3. We varied the noise level, which represents the strength of Gaussian noise adding to the target response matrix, from 0 (no noise) to 0.5 (high noise). **a** and **b** represent the performance based on different statistics: drug-averaged PCC and drug-averaged RMSE

In this section, we evaluated the performance of SRMF and compared it with KBMF [16] and DLN [17] by applying them to a set of simulated data (Additional file 3). These three methods all integrated drug similarity and cell line similarity to drug response prediction. The drug-averaged PCC and RMSE were used as metrics to assess the performance of different methods. We ran each method on simulated data and repeated this procedure for 200 times. Then the drug-averaged PCC and RMSE of 200 realizations were averaged, respectively. As shown in Fig. 3a, the drug-averaged PCC values of SRMF are still higher even though high noise levels exist. Moreover, the drug-averaged RMSE values of SRMF decrease slower than the other two approaches when the data noise increases (Fig. 3b). Thus, SRMF performs better than KBMF and DLN in the current simulation settings.

### 10-fold cross-validation on GDSC and CCLE drug response datasets

**Fig. 4:**
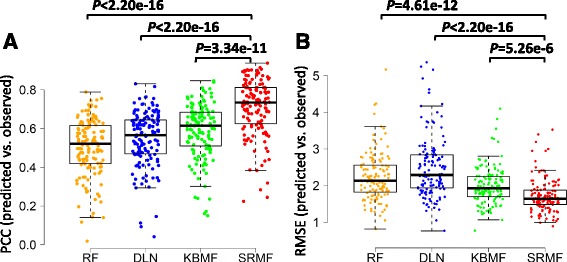
Box plots of four methods on GDSC dataset with respect to different evaluation metrics. **a** Pearson correlation coefficient between predicted and observed response values of sensitive and resistant cell lines for each drug. **b** Root mean squared error between predicted and observed drug responses of sensitive and resistant cell lines for each drug. The t-test was used to measure the statistical difference between two groups.

**Fig. 5:**
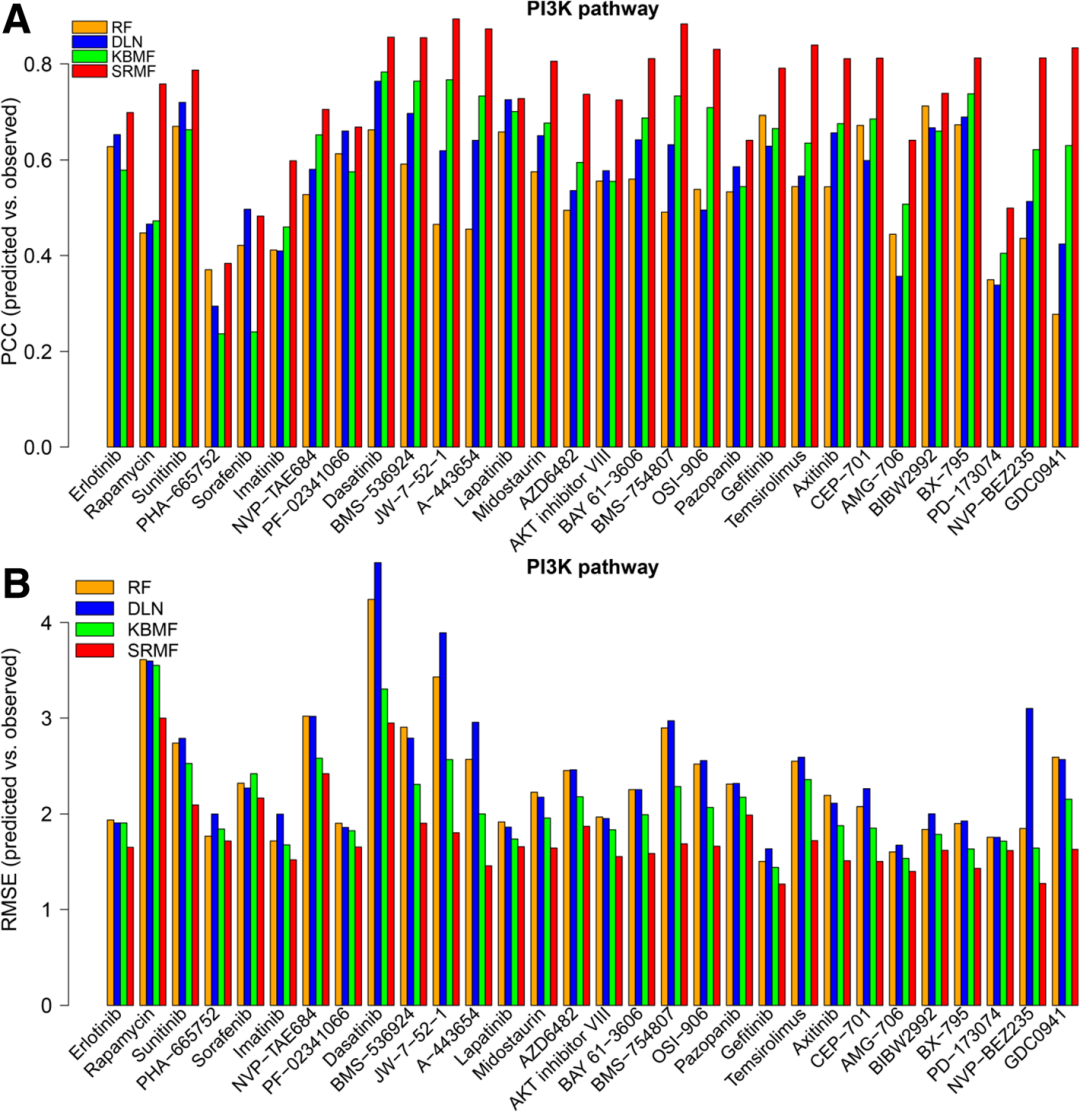
Prediction performance comparisons of four methods for the drugs targeting genes in the PI3K pathway with respect to two measurements. **a** Pearson correlation coefficient between predicted and observed response values of sensitive and resistant cell lines for each drug. **b** Root mean squared error between predicted and observed drug responses of sensitive and resistant cell lines for each drug

We conducted 10-fold cross-validation to evaluate the performance of SRMF in the GDSC dataset with IC50 as drug response measurement. The drug response entries were divided into 10 folds randomly with almost the same size. The 9-fold was used as a training set and the remaining 1-fold was used as a test set. The prediction process was repeated 10 times for each fold as a test set. Here, we compared SRMF with three state-of-the-art methods, namely, KBMF, DLN and RF [18]. Surprisingly, SRMF achieved best prediction performance with weight parameter for drug similarity*λ*_*d*_ = 0, which means that drug structure did not contribute to the prediction performance improvement of SRMF. Table 1 shows the comparison results obtained by various methods. As shown in Table 1, SRMF attains the best measure values in all metrics over the GDSC dataset. The drug-averaged PCC_S/R (Pearson correlation between predicted and observed responses of sensitive and resistant cell lines) obtained by SRMF is 0.71, which is 20.34% better than the second method KBMF. The drug-averaged RMSE_S/R (root mean square error between predicted and observed responses of sensitive and resistant cell lines) obtained by our method is 1.73, which is 13.50% lower than that obtained by the second method KBMF. Notably, the prediction performance of SRMF was decreased when the gene expression data was dropped out (setting weight parameter for cell line similarity*λ*_*c*_ = 0) (Table 1). Figure 4 shows the box plots of different methods with respect to the above two evaluation metrics for each drug. To further evaluate the prediction performance of SRMF on individual drugs, the comparison results of four models for the drugs targeting genes in the PI3K and ERK pathways are shown in Fig. 5 and Additional file 4, respectively, which indicate that SRMF obtained higher PCC and lower RMSE for most drugs.

**Table 1:** The comparison results of different methods obtained under the 10-fold cross validation on GDSC dataset

Methods	Drug-averaged PCC_S/R	Drug-averaged RMSE_S/R	Drug-averaged RMSE_S/R	Drug-averaged RMSE
SRMF (drug response + gene expression)	0.71 (±0.15)	1.73 (±0.46)	0.62 (±0.16)	1.43 (±0.36)
SRMF (drug response)	0.69 (±0.16)	1.72 (±0.48)	0.59 (±0.17)	1.45 (±0.39)
KBMF	0.59 (±0.14)	2.00 (±0.51)	0.49 (±0.14)	1.59 (±0.42)
DLN	0.55 (±0.14)	2.49 (±0.85)	0.44 (±0.13)	2.08 (±0.83)
RF	0.50 (±0.15)	2.23 (±0.66)	0.40 (±0.14)	1.69 (±0.50)

PCC_S/R—Drug-averaged Pearson correlation for responses from sensitive and resistant cell lines; RMSE_S/R—Drug-averaged root-mean-square error for responses from sensitive and resistant cell lines; PCC—Drug-averaged Pearson correlation for responses across all cell lines; RMSE—Drug-averaged root-mean-square error for responses across all cell lines. The value shown in the bracket represents standard deviation

We further validated the prediction performance of SRMF on CCLE dataset with active area as drug response measurement using the same manner. Here the low dimensionality *K* was set as 12. The comparison results of four models are shown in Table 2. SRMF also attained the best measure values in all metrics. The drug-averaged PCC_S/R obtained by SRMF is 0.78, which is 9.86% better than the second competing method DLN. The drug-averaged RMSE_S/R obtained by SRMF is 0.74, which is 6.33% lower than that achieved by the second method RF. As in the GDSC dataset, gene expression versus drug structure indeed improves the prediction performance of SRMF in CCLE dataset. Notably, one may assess treatment potential not by absolute values of drug response data, but rather by their relative order, because of batch effect of different experiments. So compared to RMSE, PCC might be a better measurement of prediction performance [4, 15, 17]. In fact, even the published original data from GDSC and CCLE have different magnitudes in IC50 for their common drugs [23]. Thus, SRMF achieved better predictive power as to Pearson correlation, suggesting that it can potentially be used in drug repositioning.

**Table 2:** The comparison results of different methods obtained under the 10-fold cross validation on CCLE dataset

Methods	Drug-averaged PCC_S/R	Drug-averaged RMSE_S/R	Drug-averaged PCC	Drug-averaged RMSE
SRMF (drug response + gene expression)	0.78 (±0.07)	0.74 (±0.23)	0.71 (±0.09)	0.57 (±0.18)
SRMF (drug response)	0.76 (±0.08)	0.75 (±0.23)	0.69 (±0.09)	0.60 (±0.23)
KBMF	0.65 (±0.10)	0.81 (±0.20)	0.71 (±0.10)	0.64 (±0.17)
DLN	0.71 (±0.06)	0.99 (±0.43)	0.64 (±0.06)	0.86 (±0.42)
RF	0.69 (±0.10)	0.79 (±0.26)	0.62 (±0.11)	0.61 (±0.20)

PCC_S/R—Drug-averaged Pearson correlation for responses from sensitive and resistant cell lines; RMSE_S/R—Drug-averaged root-mean-square error for responses from sensitive and resistant cell lines; PCC—Drug-averaged Pearson correlation for responses across all cell lines; RMSE—Drug-averaged root-mean-square error for responses across all cell lines. The value shown in the bracket represents standard deviation

### Identification of consistent and novel drug-cancer gene associations for predicted response data

**Fig. 6:**
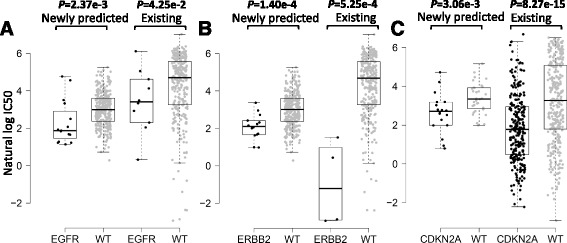
The associations of drug sensitivity and cancer gene mutations were consistent for predicted response data. **a** and **b** grouped cell line response values for lapatinib based on their EGFR mutation profiles and ERBB2 mutation profiles, respectively. WT refers to the non-mutated (wide type) cell lines. **c** grouped cell line response values for PD-0332991 based on their CDKN2A mutation profile

Using SRMF validated in the previous subsections, we trained a model on all available data and used it to predict the missing responses in the GDSC dataset. Here we focused on an EGFR and ERBB2 (also known as HER2) inhibitor lapatinib, where more than half of response values (342/652) were missing, and a cyclin D kinases (CDKs) 4 and 6 inhibitor PD-0332991, where nearly 10% of response values (62/652) were missing. There were clear associations between EGFR and ERBB2 mutations and sensitivity to lapatinib that targets the product of these genes [24, 25]. Here, we grouped the unassayed cell lines based on their EGFR mutation profiles, and found that the EGFR-mutated cell lines were significantly more sensitive to lapatinib. This prediction happened to coincide with that in assayed cell lines (Fig. 6a). Similar fact was observed with predicted response of ERBB2-mutated cell lines to lapatinib (Fig. 6b). As to PD-0332991, the predicted results show that CDKN2A-mutated cell lines are more sensitive to PD-0332991 (Fig. 6c), and this prediction was consistent with that in assayed cell lines and in agreement with previously published study [26]. In summary, even though SRMF does not specifically model mutation information, it can correctly predict consistent drug-cancer gene associations for unassayed cell lines.

**Fig. 7:**
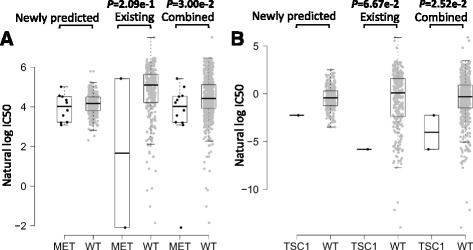
The new associations of drug sensitivity and cancer genes were identified based on a combination of newly predicted drug responses and available observations. **a** grouped cell line response values for PHA-665752 based on their MET amplification profiles. WT refers to the non-mutated (wide type) cell lines. **b** grouped cell line response values for rapamycin based on their TSC1 mutation profile

The newly predicted drug responses combined with existing drug responses were able to detect novel drug-cancer gene associations as well. For example, MET amplification was significantly associated with sensitivity to c-Met inhibitor PHA-665752 [27, 28], which was obtained by combining newly predicted drug responses and available observations versus available observations themselves (Fig. 7a), confirming the need for complementing the missing drug response values to capture new drug-sensitizing genotypes. The significant association between TSC1 mutation and sensitivity to mTOR inhibitor rapamycin [29] was identified based on a combination of newly predicted drug responses and available observations versus available observations themselves (Fig. 7b).

### Drug repositioning and novel genomic correlates of drug sensitivity

**Fig. 8:**
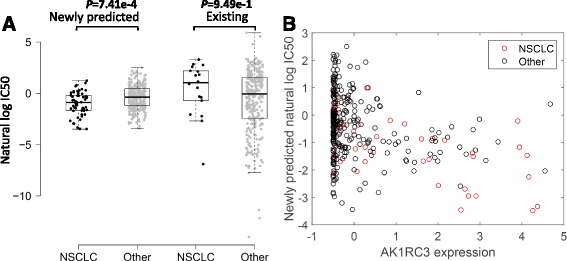
Repositioning of rapamycin and identification of a novel genomic correlate of rapamycin sensitivity. **a** grouped cell line response values for PHA-665752 based on their tissue types. NSCLC refers to the non-small cell lung cancer. **b** The scatter plot displays the association between AK1RC3 expression and newly predicted rapamycin sensitivity. *Red circles*, NSCLC cell lines; *black circles*, cell lines from other tumour types

The newly predicted drug responses of GDSC dataset can aid in drug repositioning. The mTOR inhibitor rapamycin was sensitive to non-small cell lung cancer (NSCLC) [30] based on newly predicted drug responses versus available observations (Fig. 8a). Furthermore, we applied elastic net regression, a penalized linear modelling technique, to identify genomic correlates of rapamycin sensitivity by integrating gene expression data and cell line responses to rapamycin including newly predicted response values and existing data [3–5]. Expression of AK1RC3 and HINT1 was identified as the top two sensitive signatures for rapamycin. Higher AK1RC3 expression was correlated with newly predicted sensitivity to rapamycin (Fig. 8b, Pearson correlation coefficient PCC=−0.35, *P* value=1.33 × 10^‐10^). Similar situation appeared with HINT expression (PCC=−0.24, *P* value=1.07 × 10^‐5^). Interestingly, AK1RC3 has been suggested as an adjunct marker for differentiating small cell carcinoma from NSCLC [31], and the increased expression of HINT1 inhibits the growth of NSCLC cell lines [32].

## Discussion

SRMF currently incorporated the gene expression profile based cell line similarity. Notably, SRMF can be extended to incorporate multiple types of similarity measures for cell lines through weighted low-rank approximation [20] and multiple kernel learning techniques [33]. Consequently, as to the two datasets used in the current study, some other genomic features of cell lines such as copy number variation, somatic mutation and pathways could potentially improve the performance of SRMF. Moreover, there are already some large panels of cancer cell lines for which multiple layer omics data such as microRNA expression, DNA methylation and reverse-phase protein array, and their related drug responses have been experimentally determined [5, 18, 21]. With increasing data on drug responses becoming available over time, and extended matrix factorization models to utilize the above heterogeneous data, we hope this matrix factorization based approach will have much better predictive power. Besides, our approach can be applied to other research fields such as modelling the causal regulatory network by integrating chromatin accessibility and transcriptome data in matched samples, which are deposited in Encyclopedia of DNA Elements (ENCODE) and Roadmap Epigenomic projects [34].

## Conclusions

In this study, we developed a similarity-regularized matrix factorization method SRMF to predict the response of cancer cell lines to drug treatments for IC50 values in the GDSC and activity areas in the CCLE study. The performance of SRMF was first evaluated through simulation studies and further validated by the 10-fold cross validation on GDSC and CCLE datasets. Clearly, SRMF shows better overall prediction performance than other methods in the comparison study. Finally, in comparison with existing data, the newly predicted drug responses of GDSC dataset can find consistent and novel drug-cancer gene associations and aid in drug repositioning.

## Additional files

Additional file 1: Obtaining the updating formulas of *U* and *V* by alternating minimization algorithm. The derivation process of the updating formulas is described in detail. (PDF 192 kb)

Additional file 2: The hierarchical clustering of drugs in GDSC dataset based on their PubChem fingerprint descriptors. The similarity between pair fingerprint descriptors of drugs was measured by the Jaccard coefficient. The scale to the left of the dendrogram depicts the distance value (1-Jaccard coefficient) represented by the length of the dendrogram branches connecting pairs of node. The distance threshold was specified to 0.29 to group the drugs into clusters. (PDF 9 kb)

Additional file 3: A set of simulated data used to evaluate the prediction performance of SRMF. Target drug responses, their perturbations with similarities of drugs and cell lines used as inputs for SRMF are simulated. Besides, an example for illustrating the efficiency of SRMF is described in detail. (PDF 185 kb)

Additional file 4: Prediction performance comparisons of four methods for the drugs targeting genes in the ERK pathway with respect to two measurements. A) Pearson correlation coefficient between predicted and observed response values of sensitive and resistant cell lines for each drug. B) Root mean squared error between predicted and observed drug responses of sensitive and resistant cell lines for each drug. (PDF 381 kb)

## Abbreviations

CCLE: Cancer Cell Line Encyclopedia
DLN: Dual-layer network
GDSC: Genomics of Drug Sensitivity in Cancer
KBMF: Kernelized Bayesian matrix factorization
PCC: Pearson correlation coefficient
PCC_S/R: PCC for drug responses from sensitive and resistant cell lines
RF: Random forests
RMSE: Root mean square error
RMSE_S/R: RMSE for drug responses from sensitive and resistant cell lines
SRMF: Similarity-regularized matrix factorization
