# Supplementary material for: Improved anticancer drug response prediction in cell lines using matrix factorization with similarity regularization
Source: BMC Cancer. 2017 Aug 2;17:513. doi: 10.1186/s12885-017-3500-5 (PMC5541434; doi:10.1186/s12885-017-3500-5)
Supplement: Supplementary file 1 — Obtaining the updating formulas of U and V by alternating minimization algorithm. The derivation process of the updating formulas is described in detail. (PDF 192 kb) [file 12885_2017_3500_MOESM1_ESM.pdf]

The SRMF algorithm

Here let u_i and v_j be the *i*-th and *j*-th row vectors of *U* and *V*, respectively. We take the partial derivative of objective function L, i.e. $\frac{\partial L}{\partial u_i}$ and obtain the updating rule of *U* by setting $\frac{\partial L}{\partial u_i}$=0, as follows:

$$\frac{\partial L}{\partial u_i}\{\sum_{j=1}^{n} W_{ij}\times(Y_{ij}-u_i v_j^T)]^2+\lambda_l\sum_{j=1}^{n}u_{ij}^2+\lambda_d\sum_{p=1}^{m}\sum_{q=1}^{m}[S_d(p,q)-u_p u_q^T]^2\}$$

$$=2\sum_{j=1}^{n}W_{ij}^2\times(Y_{ij}-u_i v_j^T)\times(-v_j)+2\lambda_l u_i+4\lambda_d\sum_{p=1}^{m}[S_d(p,i)-u_p u_i^T]\times(-u_p)$$

$$=2\sum_{j=1}^{n}W_{ij}^2\times(Y_{ij}-u_i v_j^T)\times(-v_j)+2\lambda_l u_i+4\lambda_d\sum_{p=1}^{m}[S_d(p,i)-u_i u_p^T]\times(-u_p)$$

$$=0$$

As W_{ij} = 0 or 1, so

$$u_i=[\sum_{j=1}^{n}W_{ij}Y_{ij}v_j+2\lambda_d\sum_{p=1}^{m}S_d(p,i)u_p](\sum_{j=1}^{n}W_{ij}v_j^T v_j+\lambda_l I_K+2\lambda_d\sum_{p=1}^{m}u_p^T u_p)^{-1} \qquad (1)$$

Similarly, according to $\frac{\partial L}{\partial b_j}$=0, we obtain the updating rule of *V* as follows:

$$v_j=[\sum_{i=1}^{m}W_{ij}Y_{ij}u_i+2\lambda_c\sum_{h=1}^{n}S_c(h,j)v_h](\sum_{i=1}^{m}W_{ij}u_i^T u_i+\lambda_l I_K+2\lambda_c\sum_{h=1}^{n}v_h^T v_h)^{-1} \qquad (2)$$
