# Supplementary figures and images for: Improved anticancer drug response prediction in cell lines using matrix factorization with similarity regularization
Source: BMC Cancer. 2017 Aug 2;17:513. doi: 10.1186/s12885-017-3500-5 (PMC5541434; doi:10.1186/s12885-017-3500-5)

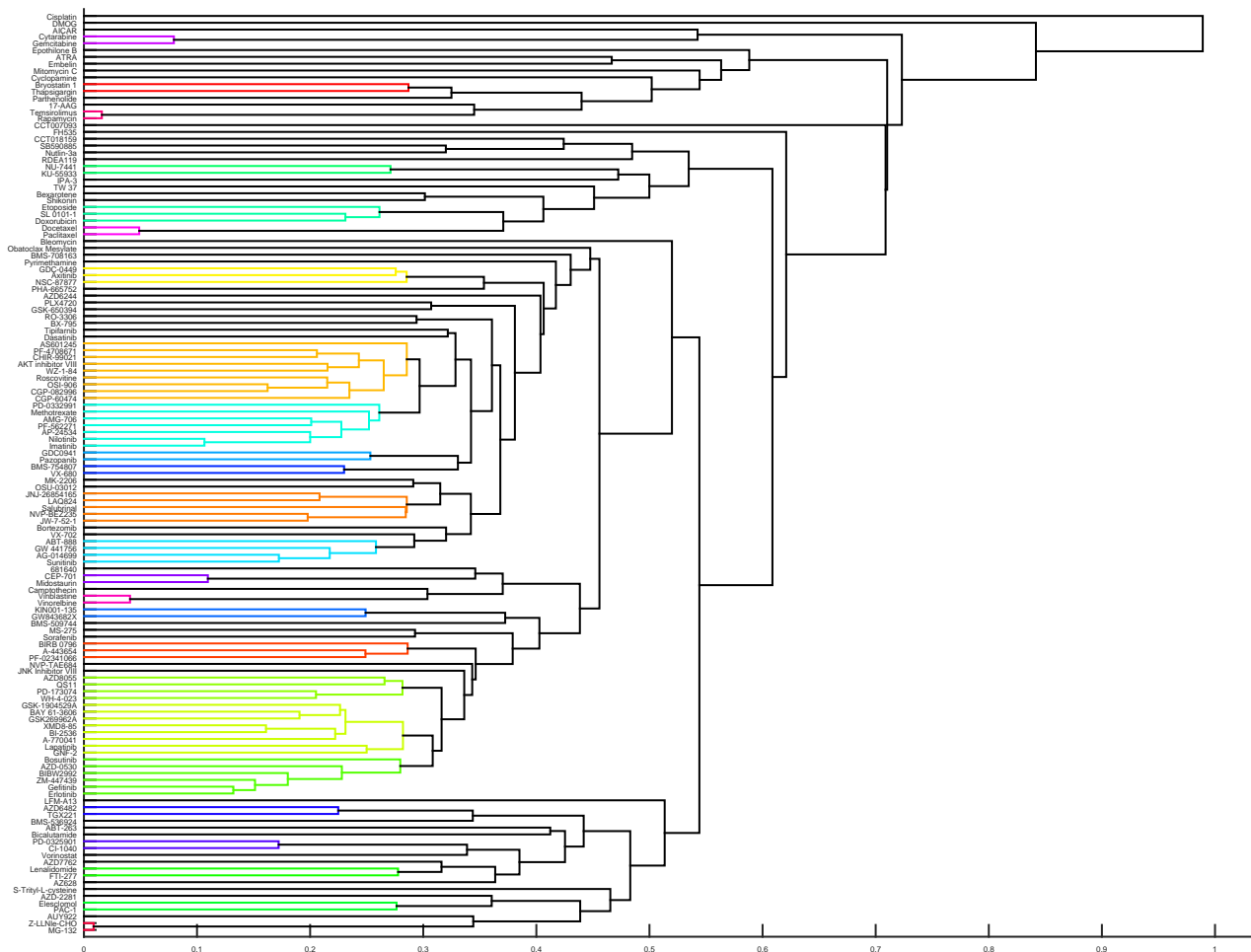

Supplement: Supplementary file 2 — The hierarchical clustering of drugs in GDSC dataset based on their PubChem fingerprint descriptors. The similarity between pair fingerprint descriptors of drugs was measured by the Jaccard coefficient. The scale to the left of the dendrogram depicts the distance value (1-Jaccard coefficient) represented by the length of the dendrogram branches connecting pairs of node. The distance threshold was specified to 0.29 to group the drugs into clusters. (PDF 9 kb) [file 12885_2017_3500_MOESM2_ESM.pdf]

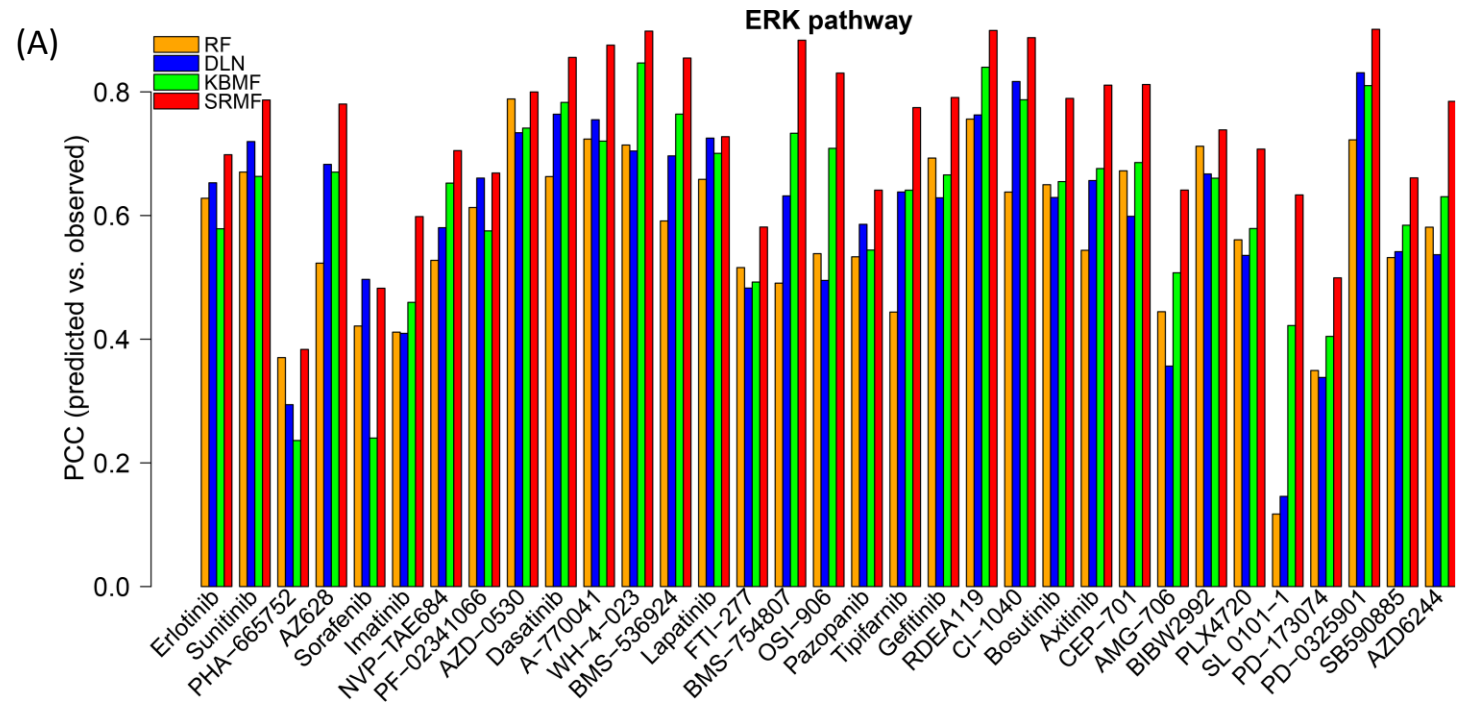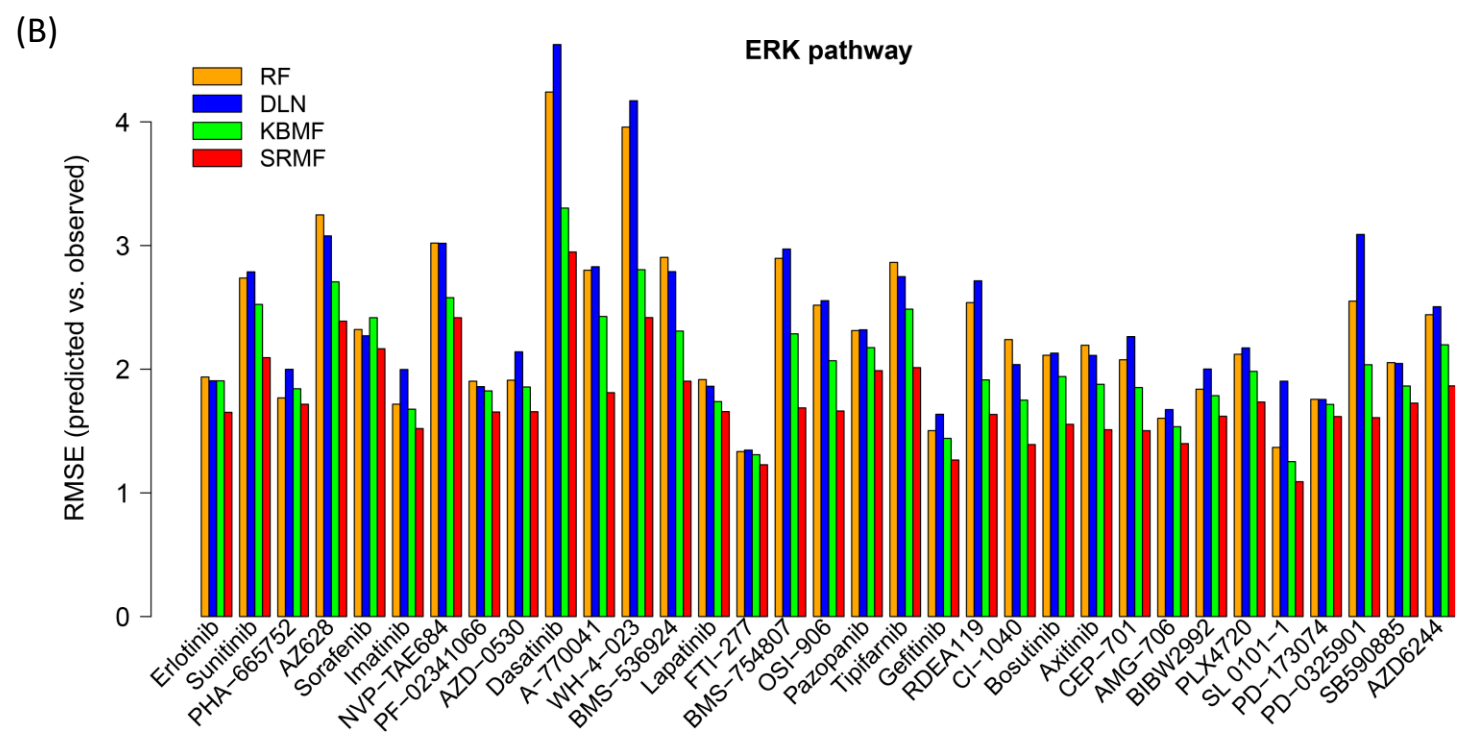

Supplement: Supplementary file 4 — Prediction performance comparisons of four methods for the drugs targeting genes in the ERK pathway with respect to two measurements. A) Pearson correlation coefficient between predicted and observed response values of sensitive and resistant cell lines for each drug. B) Root mean squared error between predicted and observed drug responses of sensitive and resistant cell lines for each drug. (PDF 381 kb) [file 12885_2017_3500_MOESM4_ESM.pdf]
