# Supplementary material for: Improved anticancer drug response prediction in cell lines using matrix factorization with similarity regularization
Source: BMC Cancer. 2017 Aug 2;17:513. doi: 10.1186/s12885-017-3500-5 (PMC5541434; doi:10.1186/s12885-017-3500-5)
Supplement: Supplementary file 3 — A set of simulated data used to evaluate the prediction performance of SRMF. Target drug responses, their perturbations with similarities of drugs and cell lines used as inputs for SRMF are simulated. Besides, an example for illustrating the efficiency of SRMF is described in detail. (PDF 185 kb) [file 12885_2017_3500_MOESM3_ESM.pdf]

Simulated data

All drug response matrices we used have 100 drugs and 150 cell lines. We first embedded five true clusters in the response matrix *Rtrue* as follows: $Rtrue = UV^T$, where *U* and *V* are shown in Table S1, and their dimensions are 100×5 and 150×5, respectively. The (x, y)-element of *Rtrue* represents activity of drug *x* to cell line *y* such as activity area, and is 0 if drug *x* is the most resistant to cell line *y*; otherwise a higher value indicates a better sensitivity of cell line *y* to drug *x*. Then Gaussian noise *E* was added to *Rtrue* with *Rnoisy*=*Rtrue*-(noise_level)*E, where noise_level is a value being changed from 0 to 0.5 by the interval of 0.05. Furthermore, we masked 50% of the values of the cluster and 12.5% of the rest of values for *Rnoisy*, and the resulting matrix was used as input for the drug responses.

As to drug (cell line) similarity, we first generated a true similarity matrix *Strue* so that the (i, j)-element of *Strue* is 1 if d_i and d_j (or c_i and c_j) belong to the same true cluster; otherwise this value is 0. We then generated a random matrix *Srandom*, where each element of this matrix randomly takes a value between 0 and 1. We finally generated similarity matrices (for both drugs and cell lines) by *Snoisy*=*Strue*-(noise_level)*Srandom, where noise_level is a value being changed from 0 to 0.5 by the interval of 0.05, and the diagonal elements of *Snoisy* are forced to be 1. Figure 1S shows a simulated example with noise level = 0.2 to illustrate the efficiency of SRMF.

U

Row\Col	1st	2nd	3rd	4th	5th
$u_1 \vdots u_{20}$	rand(20,1)	0	0	0	0
$u_{21} \vdots u_{40}$	0	rand(20,1)	0	0	0
$u_{41} \vdots u_{60}$	0	0	rand(20,1)	0	0
$u_{61} \vdots u_{80}$	0	0	0	rand(20,1)	0
$u_{81} \vdots u_{100}$	0	0	0	0	rand(20,1)

V

Row\Col	1st	2nd	3rd	4th	5th
$v_1 \vdots v_{50}$	rand(50,1)	0	0	0	0
$v_{51} \vdots v_{90}$	0	rand(40,1)	0	0	0
$v_{91} \vdots v_{120}$	0	0	rand(30,1)	0	0
$v_{121} \vdots v_{140}$	0	0	0	rand(20,1)	0
$v_{141} \vdots v_{150}$	0	0	0	0	rand(10,1)

Table S1. Detailed *U and V* used to construct target drug response matrix *Strue*. The function rand(x, y) creates an $x \times y$ dimensional matrix with random elements in the range of (0.4, 1).

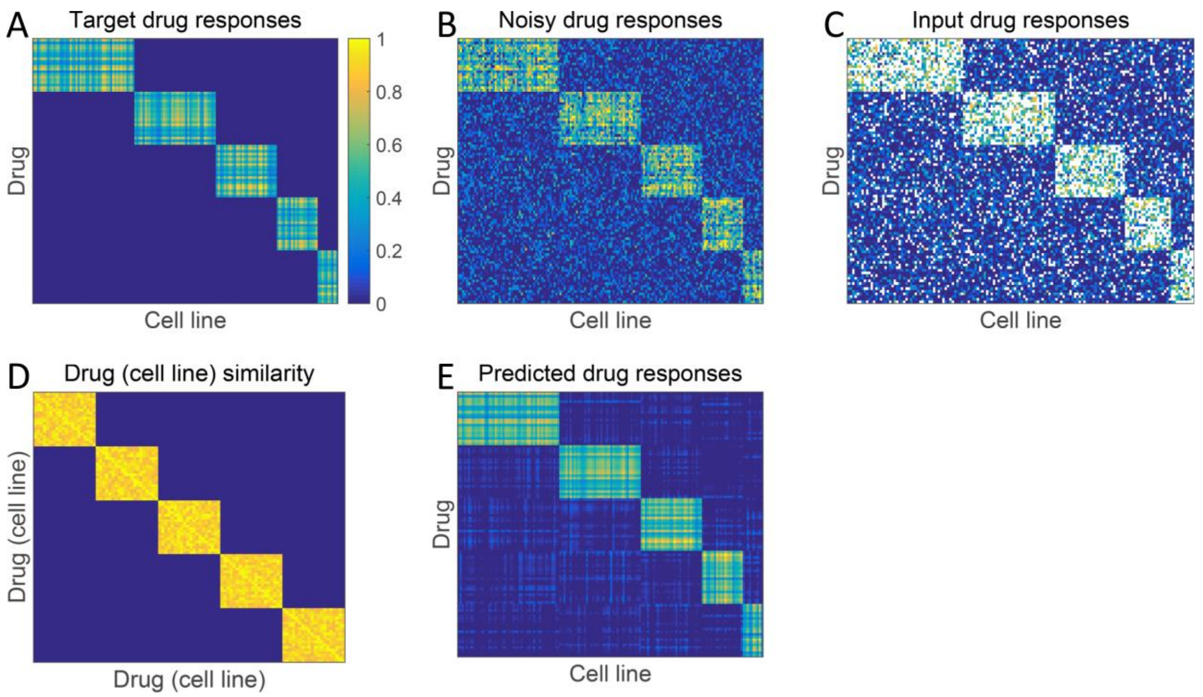

Figure S1. A simulated example illustrating the efficiency of SRMF. A) The target drug response matrix. B) The Gaussian noise with noise level = 0.2 is added to the target response matrix. C) For each drug cluster, 50% of values in the bicluster and 12.5% of the remaining cell lines were masked as missing data. D) The drug (cell line) similarity matrix. E) The predicted drug response matrix.
